# Efficacy of Janus kinase inhibitors in the treatment of acute exacerbations of rheumatoid arthritis–associated interstitial lung disease: a retrospective observational case series

**DOI:** 10.3389/fimmu.2026.1800161

**Published:** 2026-04-13

**Authors:** Momoko Okamoto, Keita Fujikawa, Tomo Mihara, Seiji Doi, Shinya Tomari, Tomohiro Koga, Takashi Kido, Noriho Sakamoto, Akinari Mizokami, Hiroshi Mukae, Atsushi Kawakami

**Affiliations:** 1 Department of Rheumatology, Japan Community Healthcare Organization, Isahaya General Hospital, Isahaya, Japan; 2 Department of Respiratory Medicine, Japan Community Healthcare Organization, Isahaya General Hospital, Isahaya, Japan; 3 Department of Immunology and Rheumatology, Graduate School of Biomedical Sciences, Nagasaki University, Nagasaki, Japan; 4 Department of Respiratory Medicine, Nagasaki University Graduate School of Biomedical Sciences, Nagasaki, Japan

**Keywords:** acute exacerbation, high-resolution computed tomography (HRCT), interstitial lung disease, JAK inhibitors, rheumatoid arthritis

## Abstract

**Background:**

Acute exacerbation of rheumatoid arthritis–associated interstitial lung disease (AE-RA-ILD) is a life-threatening condition for which no standard therapy has been established. Although Janus kinase (JAK) inhibitors are effective for treating rheumatoid arthritis (RA) and have shown potential benefit in chronic RA-ILD, evidence supporting their use in AE-RA-ILD remains extremely limited.

**Methods:**

This retrospective observational study included consecutive patients hospitalized for AE-RA-ILD who received JAK inhibitors alongside systemic glucocorticoids between January and December 2024. Clinical characteristics, laboratory data, treatment regimens, and outcomes were extracted from medical records. High-resolution computed tomography images were quantitatively evaluated at three time points—before acute exacerbation (AE), at exacerbation onset, and after treatment—using a standardized computed tomography (CT) scoring system.

**Results:**

Six patients with AE-RA-ILD were included. In five of the six cases, the JAK inhibitor was discontinued due to an adverse event prior to AE onset, with a median interval of 9 days between discontinuation and AE onset. Upadacitinib was administered to four patients, and baricitinib to two patients. Three patients received methylprednisolone pulse therapy. Respiratory status improved in all patients, as indicated by increases in the PaO_2_/FiO_2_ ratio. The total CT score increased from baseline (175.3 ± 50.3) to AE onset (247.2 ± 53.6) and subsequently decreased after treatment (220.1 ± 58.6), with notable improvement in fibrotic lesion components. All patients survived during the 3-month observation period, although four patients experienced a decline in percent predicted forced vital capacity, and three required home oxygen therapy at discharge.

**Conclusion:**

In this retrospective case series, adjunctive treatment with JAK inhibitors was associated with improvements in respiratory status and radiological findings in patients with AE-RA-ILD. These findings suggest that JAK inhibitors may represent a promising therapeutic option for this severe condition and support the need for further investigation in larger, prospective studies.

## 1 Introduction

Rheumatoid arthritis (RA) is a systemic autoimmune disease characterized by chronic synovitis and progressive joint destruction. Beyond articular manifestations, RA frequently affects extra-articular organs, among which interstitial lung disease (ILD) is one of the most severe and prognostically significant complications. RA-associated ILD (RA-ILD) is associated with significantly increased mortality compared with RA without ILD (1).

Acute exacerbation of RA-ILD (AE-RA-ILD) is a catastrophic clinical event characterized by rapid deterioration in respiratory function accompanied by newly developed bilateral pulmonary infiltrates. Previous studies have reported 3-month mortality rates ranging from 16.7% to 48.7%, underscoring the grave prognosis associated with this condition (2). Although high-dose glucocorticoids (GCs) and adjunctive immunosuppressive therapies are commonly used in clinical practice, no standardized or evidence-based treatment strategy has been established for AE-RA-ILD (3–5).

Janus kinase (JAK) inhibitors suppress multiple proinflammatory cytokine pathways by inhibiting the JAK/STAT signaling cascade and are extensively used in RA treatment (6). Emerging evidence suggests that JAK inhibitors may stabilize pulmonary involvement in chronic RA-ILD and exert therapeutic effects in other acute inflammatory lung diseases (7–9). In addition to the pathophysiological rationale for targeting the JAK/STAT pathway in alveolar epithelial cells, JAK inhibitors offer practical clinical advantages, including oral administration, a short half-life, and ease of discontinuation in the event of adverse events (10). However, evidence supporting the efficacy of JAK inhibitors in AE-RA-ILD remains extremely limited.

In this study, we conducted a retrospective observational analysis of six patients with AE-RA-ILD who were treated with JAK inhibitors alongside systemic GCs. By evaluating the clinical outcomes and radiological changes using a quantitative computed tomography (CT) scoring system, we aimed to explore the potential role of JAK inhibitors in this condition.

## 2 Materials and methods

### 2.1 Study design and patients

This retrospective observational case series included consecutive patients admitted to Isahaya General Hospital between January and December 2024 for AE-RA-ILD. All patients met the 2010 American College of Rheumatology/European League Against Rheumatism classification criteria for RA (11) and had pre-existing ILD confirmed by high-resolution computed tomography (HRCT).

Acute exacerbation (AE) was defined as worsening dyspnea within 1 month, newly developed bilateral ground-glass opacities or consolidations on HRCT, and a decline in oxygenation (a decrease in arterial PaO_2_ greater than 10 mmHg or an equivalent decline in oxygenation indices). Patients were excluded if respiratory deterioration was attributable to alternative causes, including infection, heart failure, pulmonary embolism, pneumothorax, or drug-induced lung injury (12). Drug-induced ILD (DI-ILD) has no single gold-standard diagnostic test or universally accepted scoring system (13); therefore, AE was distinguished from potential DI-ILD based on the temporal relationship to drug exposure or discontinuation, exclusion of alternative causes (particularly infection and cardiogenic pulmonary edema), and clinicoradiologic assessment. This study was approved by the Ethics Committee of Isahaya General Hospital (Approval No. 150). Written informed consent for publication was obtained from all patients.

### 2.2 Clinical and laboratory assessment

We collected clinical data before AE onset, including age, sex, RA disease duration and stage, smoking history, autoantibody status (rheumatoid factor and anticyclic citrullinated peptide antibodies), RA disease activity, percent predicted forced vital capacity (%FVC), prior disease-modifying antirheumatic drug (DMARD) use, duration of DMARD withdrawal, and suspected triggers. Post-AE data included laboratory parameters (C-reactive protein, lactate dehydrogenase, KL-6), minimum PaO_2_/FiO_2_ (P/F) ratio, maximum inspired oxygen fraction, treatment details, post-treatment %FVC, adverse events within 3 months, and clinical outcomes.

### 2.3 Radiological assessment

HRCT images obtained before AE onset, at onset, and after treatment were independently evaluated by two experienced pulmonologists certified by the Japanese Respiratory Society. They were blinded to the treatment details (including the specific JAK inhibitor) and to the timing of each CT scan (pre-onset, at onset, and post-onset). They classified CT findings into inflammatory and fibrotic components and quantified them using the CT scoring method described by Ichikado et al. (14). Final CT scores were calculated as the mean of the two evaluators’ assessments. The minimal clinically important difference for changes in HRCT scores in AE-RA-ILD has not been established. Because this study was an exploratory case series, we did not predefine a numerical threshold to classify patients as responders. Instead, changes in HRCT scores were used as the primary quantitative measure, and score reductions were interpreted in conjunction with changes in the P/F ratio, a clinical indicator of improvement in respiratory status.

### 2.4 Treatment and statistical analysis

Treatment was initiated at the discretion of the attending physicians in consultation with the pulmonologists. Continuous variables are presented as mean ± SD or median (range), as appropriate. Given the small sample size, analyses were limited to descriptive statistics.

## 3 Results

Six patients with AE-RA-ILD were treated with JAK inhibitors plus GCs. Patient characteristics before AE onset are detailed in **Table 1**. The median age was 77 years (range, 72–85), five patients were men, and the median RA disease duration was 17 years (range, 8–34). All patients had a smoking history and were positive for rheumatoid factor (RF) and anti–cyclic citrullinated peptide antibody (ACPA). HRCT showed a usual interstitial pneumonia (UIP) pattern in all cases. Five patients had previously received upadacitinib (UPA); however, all JAK inhibitors had been discontinued before AE onset because of adverse events (e.g., infection or drug eruption), with a median discontinuation period of 9 days (range, 7–98 days). Four patients exhibited possible infectious triggers before AE onset.

**Table 1:** Baseline characteristics prior to the onset of AE.

Case	Age	Sex	Duration (year)	Stage	Smoking (BI)	RF (IU/mL)	ACPA (U/mL)	SDAI	ILD pattern	%FVC before AE	DMARD before AE	Duration of DMARD withdrawal (days)	Possible triggers
1	72	F	30	III	Ever (880)	132	857.1	7.0	UIP	93.6	UPA	9	UTI
2	77	M	12	III	Ever (210)	18	14.4	2.6	UIP	72.8	UPA	8	Herpes zoster
3	85	M	34	III	Ever (560)	434	109.7	4.9	UIP	88.8	TAC	1	None
4	81	M	10	II	Ever (165)	814	61.3	1.6	UIP	90.8	UPA	7	Bacterial pneumonia
5	73	M	17	II	Ever (3000)	483	1170	0.6	UIP	105	UPA	27	Pyothorax after surgery
6	80	M	8	II	Ever (380)	100	243	0.8	UIP	94.8	UPA	98	None

BI, Brinkmann index; RF, rheumatoid factor; ACPA, anti-cyclic peptide antibody; SDAI, simplified disease activity index; ILD, interstitial lung disease; FVC, forced vital capacity; DMARD, disease-modifying anti-rheumatic drugs; AE, acute exacerbation; UIP, usual interstitial pneumonia; UTI, urinary tract infection; UPA, upadacitinib; TAC: tacrolimus.

**Figure 1:**
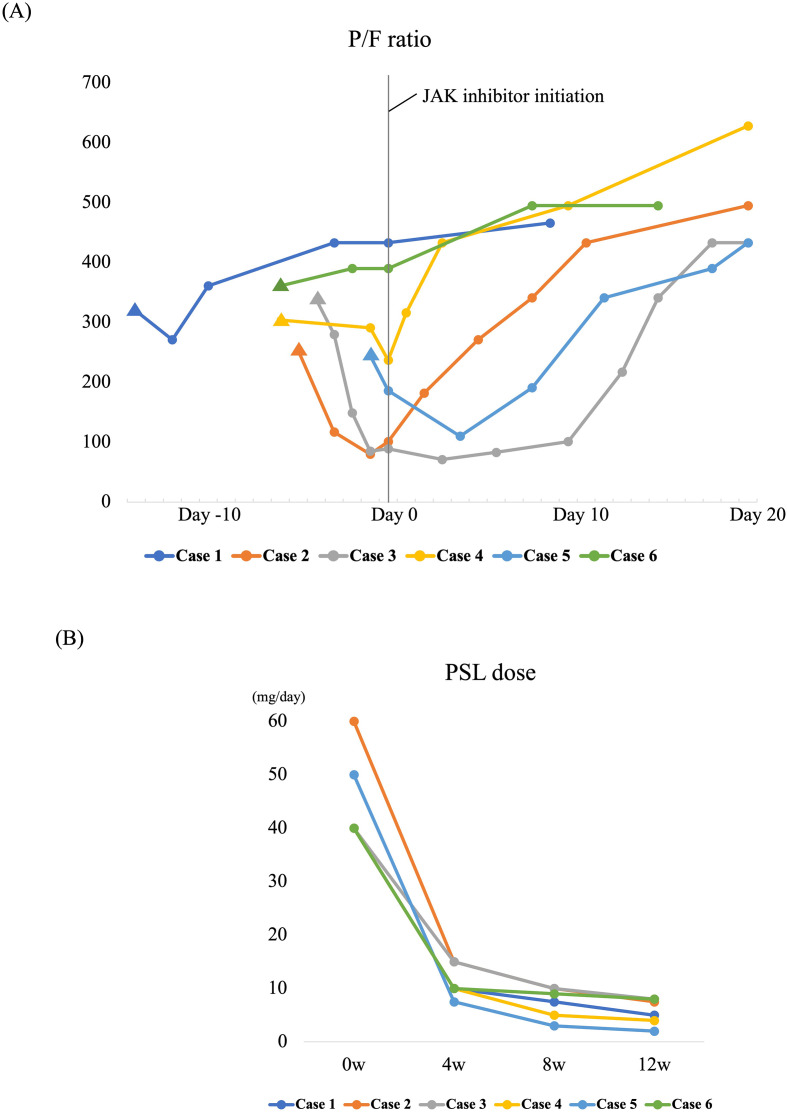
**(A)** Change in PaO_2_/FiO_2_ (P/F) ratio from onset to post-treatment of acute exacerbation of rheumatoid arthritis–associated interstitial lung disease (AE-RA-ILD). Arrowheads indicate AE at onset. **(B)** Prednisolone (PSL) tapering from treatment initiation to 3 months after AE-RA-ILD onset.

Treatment details and outcomes are presented in **Table 2**. After AE onset, oral prednisolone (PSL) was initiated at a median dose of 40 mg/day, and three patients also received methylprednisolone (mPSL) pulse therapy. Antibiotic therapy was administered in five patients. The JAK inhibitors used were UPA in four patients and baricitinib (BAR) in the other two patients; no other immunosuppressive agents or biologic therapies were administered. Three patients developed severe respiratory failure, defined as a P/F ratio ≤200, and required high-flow nasal cannula (HFNC) oxygen therapy, followed by subsequent improvement in oxygenation (**Figure 1A**). A ≥10% decline in %FVC was observed in four patients, and home oxygen therapy was initiated at discharge in three patients. The PSL dose was tapered from 40 mg/day to 12.5 mg/day at 1 month and 6.3 mg/day at 3 months (**Figure 1B**). During the 3-month observation period after treatment initiation, one patient developed bacterial pneumonia; however, no cases of herpes zoster, cytomegalovirus infection, or other apparent viral infections were observed. All six patients survived during the observation period.

**Table 2:** Clinical features, treatment, and outcome after the onset of AE.

Clinical features					Treatment						Outcome				
Case	CRP (mg/dl)	LDH (U/l)	KL-6 (U/ml)	FIO_2_ (max)	Respira-tory support	PSL (mg/day) [mg/kg]	mPSL pulse	JAKi (mg)	Days from AE onset to JAKi start	Concomitantuse of antibiotics	SDAI after 3 months	%FVC after AE	HOT at discharge	Adverse events within 3 months	Outcome
1	21.1	266	298	28	None	40 [0.8]	None	UPA 7.5	14	None	10.1	89.9	None	None	Survive
2	16.6	271	1331	80	HFNC	60 [0.9]	Yes	BAR 2	7	TAZ/PIPC	2.2	55.1	2L	None	Survive
3	16.2	193	448	55	HFNC	40 [0.7]	Yes	UPA 7.5	2	TAZ/PIPC	4.5	67.9	3L	None	Survive
4	7.0	419	863	32	None	40 [0.7]	None	BAR 2	2	SBT/ABPC	2.1	91.1	None	Bacterial pneumonia	Survive
5	17.2	195	2045	55	HFNC	50 [0.r8]	Yes	UPA 15	2	TAZ/PIPC	0.5	77.6	1L	None	Survive
6	18.8	397	222	21	None	40 [0.6]	None	UPA 7.5	7	CTRX, AZM	0.7	66.4	None	None	Survive

CRP, C-reactive protein; LDH, lactate dehydrogenase; KL-6, Krebs von den Lungen-6; FiO_2_ (max), maximum fraction of inspired oxygen; PSL, prednisolone; mPSL, methylprednisolone; JAKi, Janus kinase inhibitor; AE, acute exacerbation; SDAI, simplified disease activity index; FVC, forced vital capacity; HOT, home oxygen therapy; HFNC, high-flow nasal cannula; UPA, upadacitinib; BAR, baricitinib; TAZ/PIPC, tazobactam/piperacillin; SBT/ABPC, sulbactam/ampicillin; CTRX, ceftriaxone; AZM, azithromycin.

**Figure 2:**
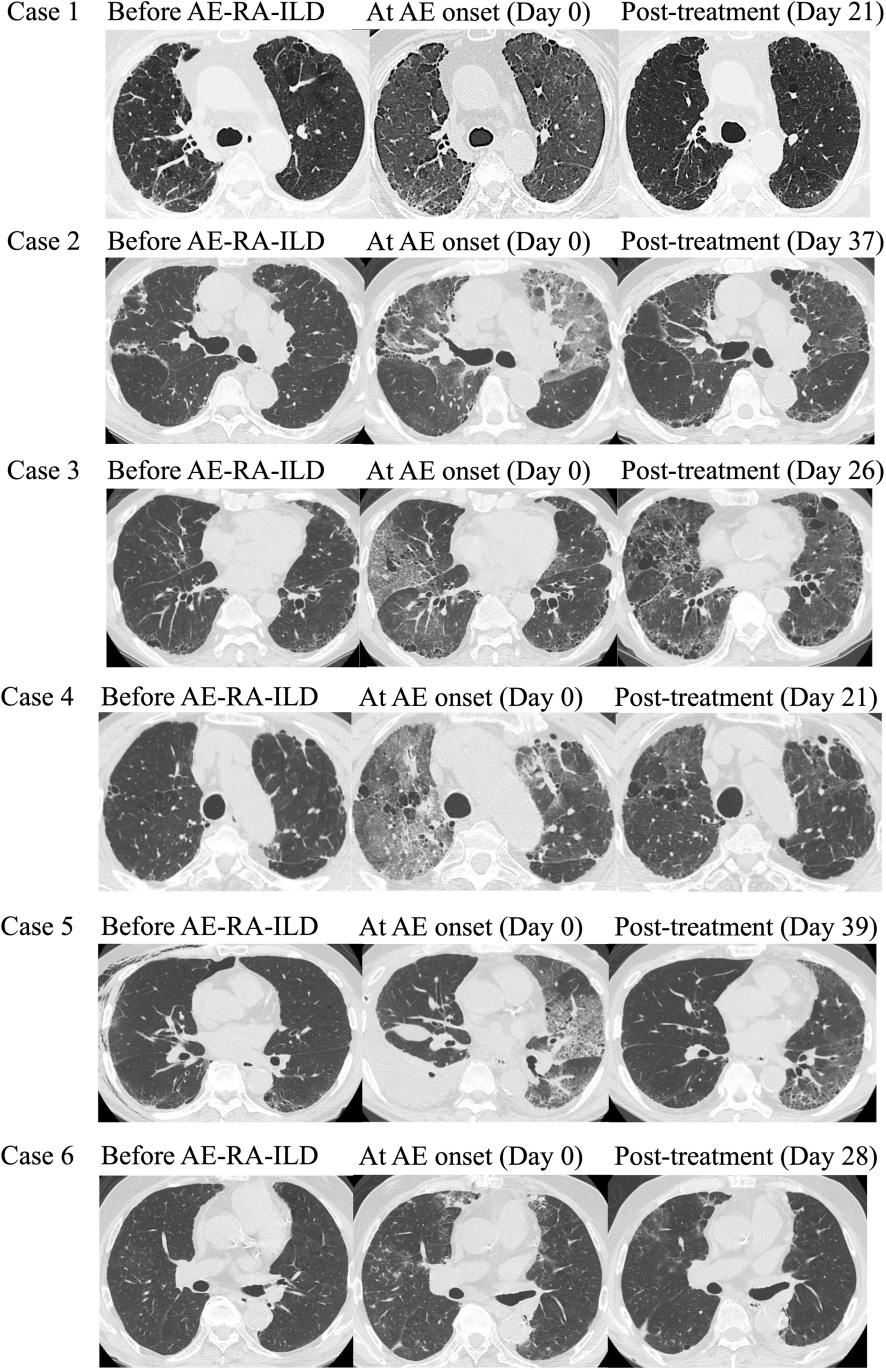
High-resolution computed tomography (HRCT) findings before rheumatoid arthritis–associated interstitial lung disease (AE-RA-ILD) onset, at exacerbation onset, and post-treatment.

HRCT findings (**Figure 2**) showed newly developed ground-glass opacities at AE onset in all patients, occasionally accompanied by consolidation or pleural effusion, which improved after treatment. CT scores (**Table 3**) worsened at AE onset across all components except honeycombing and partially improved after treatment. The total CT score increased from 175.3 ± 50.3 before AE to 247.2 ± 53.6 at onset and decreased to 220.1 ± 58.6 after treatment.

**Table 3:** Changes in the extent of HRCT findings before, at, and after AE onset.

CT finding	Before onset (n = 6)	At onset (n = 6)	After onset (n = 6)
Spared area	80.3 ± 11.7	48.3 ± 15.1	51.5 ± 24.9
Ground-glass attenuation	2.8 ± 1.9	15.6 ± 6.8	23.6 ± 15.7
Air-space consolidation	0.1 ± 0.3	2.1 ± 2.7	0.1 ± 0.3
Total area without traction bronchiolectasis or bronchiectasis	2.9 ± 1.9	17.6 ± 4.6	23.8 ± 16.0
Ground-glass attenuation plus traction bronchiolectasis or bronchiectasis	5.6 ± 2.0	18.9 ± 4.9	13.6 ± 5.3
Air-space consolidation plus traction bronchiolectasis or bronchiectasis	0.7 ± 1.7	4.9 ± 4.6	0.1 ± 0.3
Honeycombing	10.6 ± 8.4	10.3 ± 6.5	11.0 ± 7.2
Total area with traction bronchiolectasis or bronchiectasis or honeycombing	16.8 ± 10.3	34.0 ± 13.0	24.7 ± 11.3
CT score	175.3 ± 50.3	247.2 ± 53.6	220.1 ± 58.6

Data are expressed as the mean ± SD of the percentage of lung involvement. CT, computed tomography; HRCT, high-resolution computed tomography; AE, acute exacerbation.

## 4 Discussion

Approximately 10%–19% of patients with RA develop ILD (15). Although the precise pathogenesis of RA-ILD remains unclear, several risk factors have been identified, including older age, male sex, race, smoking history, exposure to air pollution, autoantibody positivity (RF and ACPA), genetic susceptibility, and high RA disease activity (15). Persistent epithelial injury in the lungs activates immune cells such as neutrophils, dendritic cells, and macrophages, thereby promoting pulmonary fibrosis through fibroblast-to-myofibroblast transition and epithelial–mesenchymal transition. The JAK/STAT and PI3K/Akt signaling pathways further contribute to fibrosis by regulating inflammatory cytokine production (15). Among patients with RA-ILD treated with JAK inhibitors, 64.5–86.1% reportedly maintain radiological stability on HRCT (7–9).

The reported risk factors for AE in RA-ILD include advanced age, male sex, smoking history, reduced %FVC, and a UIP pattern on HRCT (16). Currently identified triggers include infection, mechanical stress, microaspiration, and drug-induced lung injury (12). In our study, all six patients presented with multiple risk factors, and four had infectious episodes before AE onset. Notably, in five of the six cases, JAK inhibitor therapy had been discontinued prior to AE onset. Three of these patients (Cases 1, 2, and 4) developed AE within 9 days of discontinuation. Type I JAK inhibitors can paradoxically promote accumulation of activation-loop phosphorylated JAK while suppressing downstream STAT signaling; rapid drug withdrawal may unmask this stored signal and trigger an acute rebound in STAT phosphorylation (17). This withdrawal phenomenon has been mechanistically demonstrated for ruxolitinib in myelofibrosis, where activation-loop pJAK2 accumulation correlated with rebound STAT activation after washout. Clinically, in a series of myelofibrosis patients, the median time from ruxolitinib discontinuation to discontinuation syndrome was 7 days (range, 2–21 days) (18). Ex vivo data in rheumatoid arthritis further suggest that NK cells from patients treated with Type I JAK inhibitors become more pro-inflammatory after drug withdrawal compared with those from methotrexate-treated patients, supporting the concept of transient withdrawal-associated immune activation (19). Although discontinuation of the antifibrotic agent nintedanib has been associated with AE onset (20), no previous studies have demonstrated a link between JAK inhibitor withdrawal and AE occurrence. Based on our findings, we propose that abrupt discontinuation of JAK inhibitors might contribute to AE onset; however, this observation remains speculative and warrants further investigation.

The pathophysiology of AE-RA-ILD remains poorly understood. In CTD-ILD, including RA-ILD, serum levels of interleukin (IL)-6, IL-8, and tumor necrosis factor-α (TNF-α) are significantly higher than in patients without ILD (21). These cytokine levels are further elevated during AE-CTD-ILD, and IL-6 has been identified as an independent predictor of AE onset. IL-6 signals directly through the JAK/STAT pathway, whereas IL-8 and TNF-α do not directly utilize this pathway but contribute to secondary amplification of inflammatory responses (22). In anti-MDA5 antibody–positive dermatomyositis–associated ILD, elevated IL-6 levels (≥13.41 pg/mL) are associated with the development and poor prognosis of RP-ILD (23). Furthermore, in COVID-19 pneumonia, BAR exerts antiviral effects by inhibiting Numb-associated kinases and anti-inflammatory effects by suppressing cytokine signaling involving IL-2, IL-6, IL-10, IFN-γ, and G-CSF, thereby mitigating the cytokine storm (24). Collectively, these findings suggest that inhibition of the JAK/STAT pathway may attenuate the excessive cytokine activation underlying AE-RA-ILD.

Currently, no standardized therapy exists for AE-RA-ILD, and high-dose corticosteroids combined with antibiotics are commonly used in clinical practice (12). For steroid-refractory cases, immunosuppressants such as cyclophosphamide (CY), tacrolimus, and cyclosporine may be added. However, the efficacy of CY remains controversial, with some studies showing no survival benefit (4, 25). Reports describing the use of JAK inhibitors in AE-RA-ILD are minimal; to date, only one case has been reported in which BAR (4 mg/day), initiated on day 21 after AE onset, resulted in clinical improvement after failure of mPSL pulse therapy, tacrolimus, and CY (26). In our study, JAK inhibitors (UPA or BAR) were initiated 2–14 days after AE onset at the discretion of attending physicians. Despite three patients presenting with severe respiratory failure requiring HFNC oxygen therapy, all patients demonstrated improvement in the P/F ratio following treatment initiation. Although most patients experienced a decline in %FVC and three required home oxygen therapy at discharge, all six survived through the 3-month observation period. Clinical improvement occurred relatively rapidly after initiation of a JAK inhibitor, allowing early tapering of corticosteroids.

To assess treatment responsiveness, we conducted radiological evaluation using the CT scoring system. Ichikado et al. demonstrated that a CT score ≥210 in acute respiratory distress syndrome (ARDS) is associated with increased 60-day mortality, with higher scores for ground-glass opacities or consolidation accompanied by traction bronchiectasis observed in nonsurvivors (14). In AE of idiopathic pulmonary fibrosis (AE-IPF), a CT score ≥245 has similarly been associated with reduced survival, and the presence of ground-glass opacities with traction bronchiectasis and honeycombing has been linked to mortality (27). These findings suggest that fibrotic, rather than purely inflammatory, CT lesions are stronger predictors of poor prognosis. In our study, total CT scores at AE onset fell within ranges previously associated with unfavorable outcomes. After treatment, fibrotic lesion components—specifically ground-glass opacities with traction bronchiectasis and consolidation—showed improvement. The observed increase in ground-glass opacity scores may reflect a recovery phase from consolidation or may be attributed to the timing of HRCT acquisition, which may not have captured the point of maximal disease severity. Although the applicability of CT scoring to AE-RA-ILD warrants further investigation given its pathophysiological differences from ARDS and AE-IPF, improvement in fibrotic lesions may have contributed to the favorable outcomes observed in this case series.

One patient developed bacterial pneumonia within 3 months, but no other adverse events were observed. However, given the short observation period, the long-term safety of JAK inhibitors remains uncertain. The ORAL Surveillance study reported an increased risk of lung cancer among patients with RA aged 50 years or older who had cardiovascular risk factors and were treated with tofacitinib, compared with those receiving TNF inhibitors (28). As RA-ILD is associated with increased lung cancer risk and lung-cancer-related mortality (29), careful long-term monitoring is required. Moreover, multicenter studies with larger sample sizes are needed to establish the long-term safety and efficacy of JAK inhibitors in AE-RA-ILD.

This study has several notable limitations. First, this investigation is an uncontrolled case series without a comparison group receiving standard therapy alone (corticosteroids and antibiotics without JAK inhibitors). Therefore, we cannot conclusively attribute the observed improvements to JAK inhibitor therapy. The clinical course of AE-RA-ILD is heterogeneous, and spontaneous improvement may occur in some patients receiving intensive supportive care and corticosteroids. Although historical data reporting 3-month mortality rates of 16.7–48.7% suggest potential benefit (2), direct comparisons are limited by differences in patient characteristics, treatment strategies, and eras of care. Second, the retrospective study design introduces potential selection bias, as JAK inhibitors were initiated at the discretion of the treating physician. Third, because three patients developed AE shortly after discontinuation of upadacitinib, this observational study cannot determine whether clinical improvement following JAK inhibitor re-initiation reflects a genuine treatment effect on AE or merely suppression of a withdrawal-associated rebound phenomenon. Fourth, heterogeneity in treatment timing (2–14 days after AE onset), JAK inhibitor type and dose, and concomitant therapies (mPSL pulse therapy in three patients and antibiotics in five) limits the ability to isolate the specific contribution of JAK inhibitors. Fifth, it remains uncertain whether the reduction in the overall CT score truly reflects improvement of the underlying interstitial disease or merely resolution of superimposed inflammatory changes that may respond to high-dose corticosteroid pulse therapy, independent of JAK inhibitor use.

## 5 Conclusion

In this retrospective case series, adjunctive JAK inhibitor treatment was associated with improvements in respiratory status and radiological findings in patients with AE-RA-ILD. Although causality cannot be established, these findings suggest that JAK inhibitors may represent a promising therapeutic option for this condition, warranting further investigation in larger, controlled studies.

## Data Availability

The datasets presented in this study can be found in online repositories. The names of the repository/repositories and accession number(s) can be found in the article/supplementary material.
